# Glycogen storage disease type III: diagnosis, genotype, management, clinical course and outcome

**DOI:** 10.1007/s10545-016-9932-2

**Published:** 2016-04-22

**Authors:** Christiaan P. Sentner, Irene J. Hoogeveen, David A. Weinstein, René Santer, Elaine Murphy, Patrick J. McKiernan, Ulrike Steuerwald, Nicholas J. Beauchamp, Joanna Taybert, Pascal Laforêt, François M. Petit, Aurélie Hubert, Philippe Labrune, G. Peter A. Smit, Terry G. J. Derks

**Affiliations:** 1 Section of Metabolic Diseases, Beatrix Children’s Hospital, University of Groningen, University Medical Center Groningen, PO Box 30 001, 9700 RB Groningen, The Netherlands; 2 Glycogen Storage Disease Program, University of Florida, Gainesville, FL USA; 3 Department of Paediatrics, University Medical Centre Hamburg-Eppendorf, Hamburg, Germany; 4 Charles Dent Metabolic Unit, National Hospital for Neurology and Neurosurgery, London, UK; 5 Birmingham Children’s Hospital, Birmingham, UK; 6 Department of Occupational and Public Health (DFAA), Tórshavn, Faroe Islands; 7 Sheffield Diagnostic Genetics Service, Sheffield Children’s NHS Foundation Trust, Sheffield, UK; 8 Department of Metabolic Diseases, Children’s Memorial Health Institute, Warsaw, Poland; 9 Centre de Référence de Pathologie, Neuromusculaire Paris-Est, Institut de Myologie, GH Pitié-Salpêtrière, Assistance Publique-Hôpitaux de Paris, Paris, France; 10 Department of Genetics and Cytogenetics, AP-HP, Antoine Béclère University Hospital, University Paris Sud, Paris, France; 11 APHP, Hôpitaux Universitaires Paris Sud, hôpital Antoine Béclère, Centre de Référence des Maladies héréditaires du Métabolisme Hépatique, and Paris Sud University, Clamart, France

## Abstract

Glycogen storage disease type III (GSDIII) is a rare disorder of glycogenolysis due to *AGL* gene mutations, causing glycogen debranching enzyme deficiency and storage of limited dextrin. Patients with GSDIIIa show involvement of liver and cardiac/skeletal muscle, whereas GSDIIIb patients display only liver symptoms and signs. The International Study on Glycogen Storage Disease (ISGSDIII) is a descriptive retrospective, international, multi-centre cohort study of diagnosis, genotype, management, clinical course and outcome of 175 patients from 147 families (86 % GSDIIIa; 14 % GSDIIIb), with follow-up into adulthood in 91 patients. In total 58 *AGL* mutations (non-missense mutations were overrepresented and 21 novel mutations were observed) were identified in 76 families. GSDIII patients first presented before the age of 1.5 years, hepatomegaly was the most common presenting clinical sign. Dietary management was very diverse and included frequent meals, uncooked cornstarch and continuous gastric drip feeding. Chronic complications involved the liver (hepatic cirrhosis, adenoma(s), and/or hepatocellular carcinoma in 11 %), heart (cardiac involvement and cardiomyopathy, in 58 % and 15 %, respectively, generally presenting in early childhood), and muscle (pain in 34 %). Type 2 diabetes mellitus was diagnosed in eight out of 91 adult patients (9 %). In adult patients no significant correlation was detected between (non-) missense *AGL* genotypes and hepatic, cardiac or muscular complications. This study demonstrates heterogeneity in a large cohort of ageing GSDIII patients. An international GSD patient registry is warranted to prospectively define the clinical course, heterogeneity and the effect of different dietary interventions in patients with GSDIII.

**Electronic supplementary material:**

The online version of this article (doi:10.1007/s10545-016-9932-2) contains supplementary material, which is available to authorized users.

## Introduction

Glycogen storage disease type III (GSDIII; OMIM #232400) is a rare inborn error of glycogen degradation with an incidence of 1:100,000 (Dagli et al 2010; Kishnani et al 2010; Laforêt et al 2012). GSDIII is caused by mutations in the *AGL* gene and the subsequent deficiency of the glycogen debranching enzyme (GDE; EC no. 3.2.1.33 and 2.4.1.25, UniProt P35573). GDE contains two catalytic centres that catalyse one of the last steps in the conversion of glycogen to glucose-1-phosphate.

Patients with GSDIII present clinically with hepatomegaly, failure to thrive and fasting intolerance, biochemically associated with ketotic hypoglycaemia. Phenotypically, patients can be further classified into having either GSDIIIa (±85 %), with involvement of the liver, heart and skeletal muscle, or GSDIIIb (±15 %), in which only the liver is affected (Shen et al 1996; Laforêt et al 2012). Dietary management aims to maintain normoglycaemia and prevent hyperketonaemia by dividing sufficient carbohydrate intake throughout the day, and using additional protein as a substrate for gluconeogenesis (as recently reviewed by Derks and Smit 2015). During long-term follow-up the clinical focus shifts to the prevention and management of progressive hepatic, cardiac and myopathic complications (Dagli et al 2009; Sentner et al 2012; Verbeek et al 2015).

Current knowledge on the clinical course and outcome has been based on case reports and small single centre cohort studies, of mainly young patients, on which the current management guidelines are based (Dagli et al 2010; Kishnani et al 2010). The International Study on GSDIII (ISGSDIII) is a descriptive, retrospective, international, multi-centre cohort study on the diagnosis, genotype, management, clinical course and outcome in 175 patients with GSDIII, with follow-up into adulthood in 91 patients.

## Methods

The Medical Ethical Committee of the University Medical Centre Groningen, the Netherlands approved the study protocol (ref.no. METc2008.035). Patients were included from 17 metabolic centres in ten countries. Between 2007 and 2011 data on GSDIII patients were collected using a case record form (CRF) for every patient and anonymously archived in a database. The CRF was based on the European Study on GSDI (ESGSDI; Rake et al 2002), and modified for ISGSDIII by two authors (GPAS, CPS) (for the complete CRF, see Supplemental data 1). The CRFs were filled out either by the treating physician or by one single investigator (CPS).

GSDIII patients were included when an enzyme assay and/or *AGL* molecular analysis had confirmed the diagnosis. GSDIIIa was defined as (a) deficient GDE activity in muscle or (b) clinical and/or biochemical signs of cardiac and/or skeletal muscular involvement. Based on the family history, individual patients could be categorized as proband, symptomatic sibling or neonatally screened patient due to an affected older sibling. To study the relationship between *AGL* genotypes and GSDIII phenotypes, statistical analyses were only performed in adult patients. Cardiac involvement was defined as the presence of abnormalities corresponding to cardiac hypertrophy in the electrocardiographic and/or echocardiographic investigations. Cardiomyopathy was defined as the presence of cardiac hypertrophy in combination with 1) (severe) exercise intolerance (and/or 2) the use of pharmacological treatment for (symptoms of) heart failure.

*AGL* mutations were grouped according to the type of mutation, i.e. missense or non-missense *AGL* genotypes. Non-missense mutations resulting in either frameshift or splicing modifications were assumed to be pathogenic. Pathogenicity of novel missense mutations was predicted by five methods: Alamut Version 2.2 (©Interactive Biosoftware), PolyPhen-2 (http://genetics.bwh.harvard.edu/pph2/), SIFT (http://sift.bii.a-star.edu.sg/), whether the mutation was located in the catalytic site (exon 6, 13-15, 26-27) or an exon encoding the glycogen binding domain (exons 31-34), and the NHLBI Exome Sequencing Project (ESP) Exome Variant Server (http://evs.gs.washington.edu/EVS/).

Data were processed with IBM® SPSS® Statistics Version 20 (SPSS Inc., Chicago, Il, USA). The results were expressed as median (range) for non-parametric data, and mean (standard deviation) for parametric data. Differences between normally distributed continuous data were analysed using the unpaired two-tailed T-test. Not normally distributed data were analysed using the Mann-Whitney-U or Kruskal-Wallis test. For dichotomous data, the Fisher’s exact test was used. The level of significance was set at p < 0.05.

## Results

Two hundred and twenty-eight patients with GSDIII were identified. Data from 53 patients were incomplete and not included in the analysis. Table 1 presents demographic and diagnostic information on 175 patients with GSDIII from 147 families, of whom 91 (52 %) had reached adulthood.

**Table 1:** Demographic and diagnostic information of GSDIIIa and GSDIIIb patients

	GSDIIIa	GSDIIIb	Total
Demographic information			
Male/female (n (%))	69 (46 %)/82 (54 %)	13 (52 %)/11 (48 %)	82 (47 %)/93 (53 %)
Age (yrs: median (range))	20.6 (1–64.1)	16.4 (0.3–50.7)	19.3 (0.3–64.2)
Follow-up (yrs: median (range))	17.4 (0.5–61.1)	14.1(0.2 – 48.7)	16.2 (0.2–61.2)
Ethnicity (n)			
Asian	2	1	3
Caucasian-Mediterranean	125	23	148
African-Caribbean	7	0	7
Mixed	17	0	17

### Clinical course and presentation until establishment of the diagnosis

#### Pregnancy, birth and presenting symptoms

Post-natal hypoglycaemia was documented for six term, normal birth-weight patients (3 %). In the 147 probands, the median age at the first clinical presentation in GSDIIIa and GSDIIIb was at 0.7 year (range: day 1–8.1 years) and 1.0 year (range: day 1–6.0 years), respectively. Common presenting symptoms included hepatomegaly (98 %), hypoglycaemia (53 %), failure to thrive (49 %) and recurrent illness and/or infections (17 %).

#### Mutation analysis

**Fig. 1:**
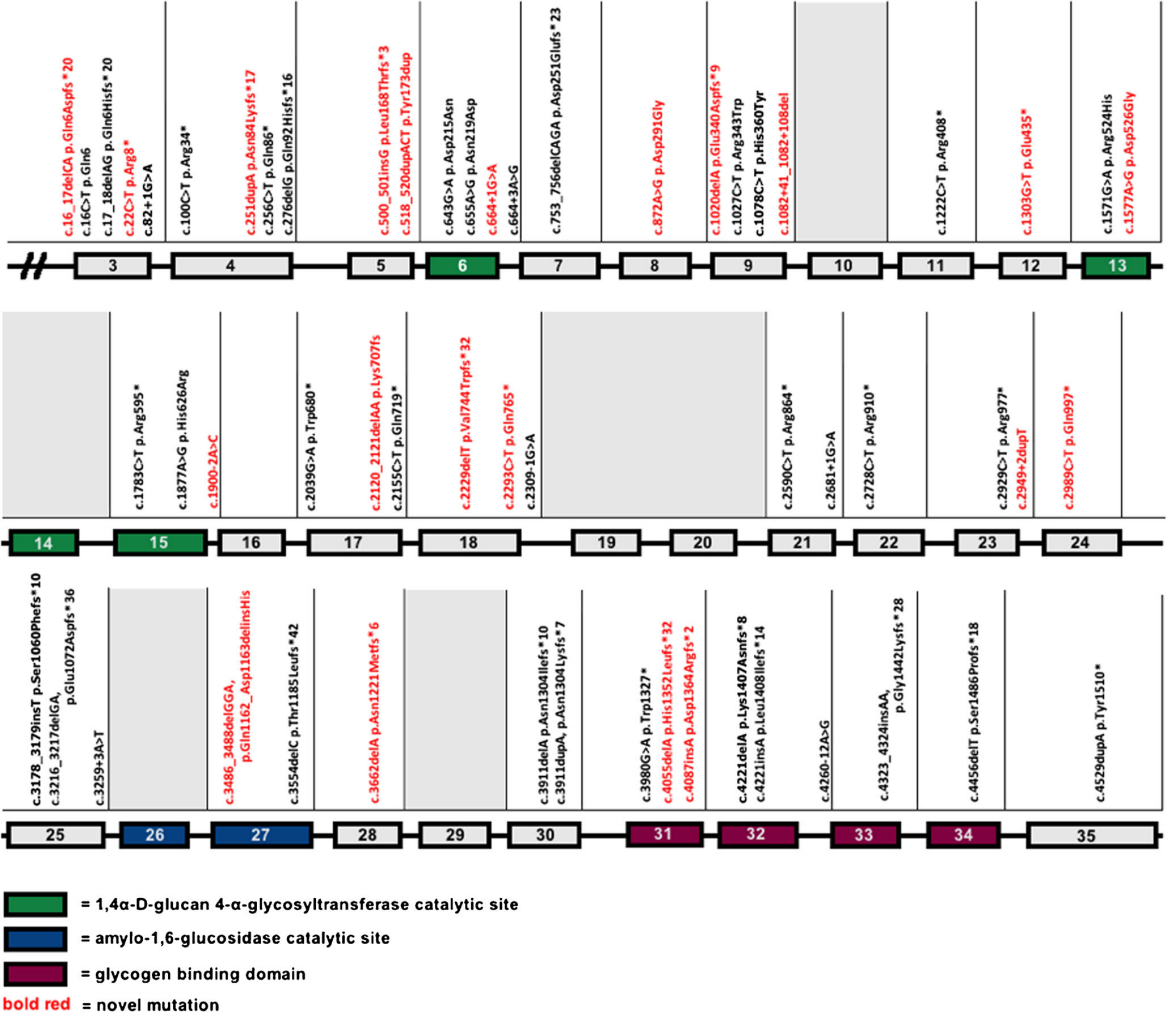
*AGL* mutations in the ISGSDIII-cohort depicted per exon/intron

Figure 1 presents the 58 individual reported mutations, including 21 novel mutations, depicted per exon/intron. *AGL* gene mutation analysis was performed in 76 out of 147 families, two causative mutations were identified in 72 families (95 %), in four families one allelic mutation was identified. The majority (50/58; 86 %) were non-missense mutations resulting in a truncated protein. Among the probands in whom both affected alleles were identified, 47 were found to be homozygotes, and 23 were compound heterozygotes. No statistically significant correlation was recognized between a (non-) missense *AGL* genotype and the occurrence of major complications (hepatic, cardiac, myopathic) in 49 adult (≥18 years) patients.

### Clinical course after establishment of the diagnosis

**Fig. 2:**
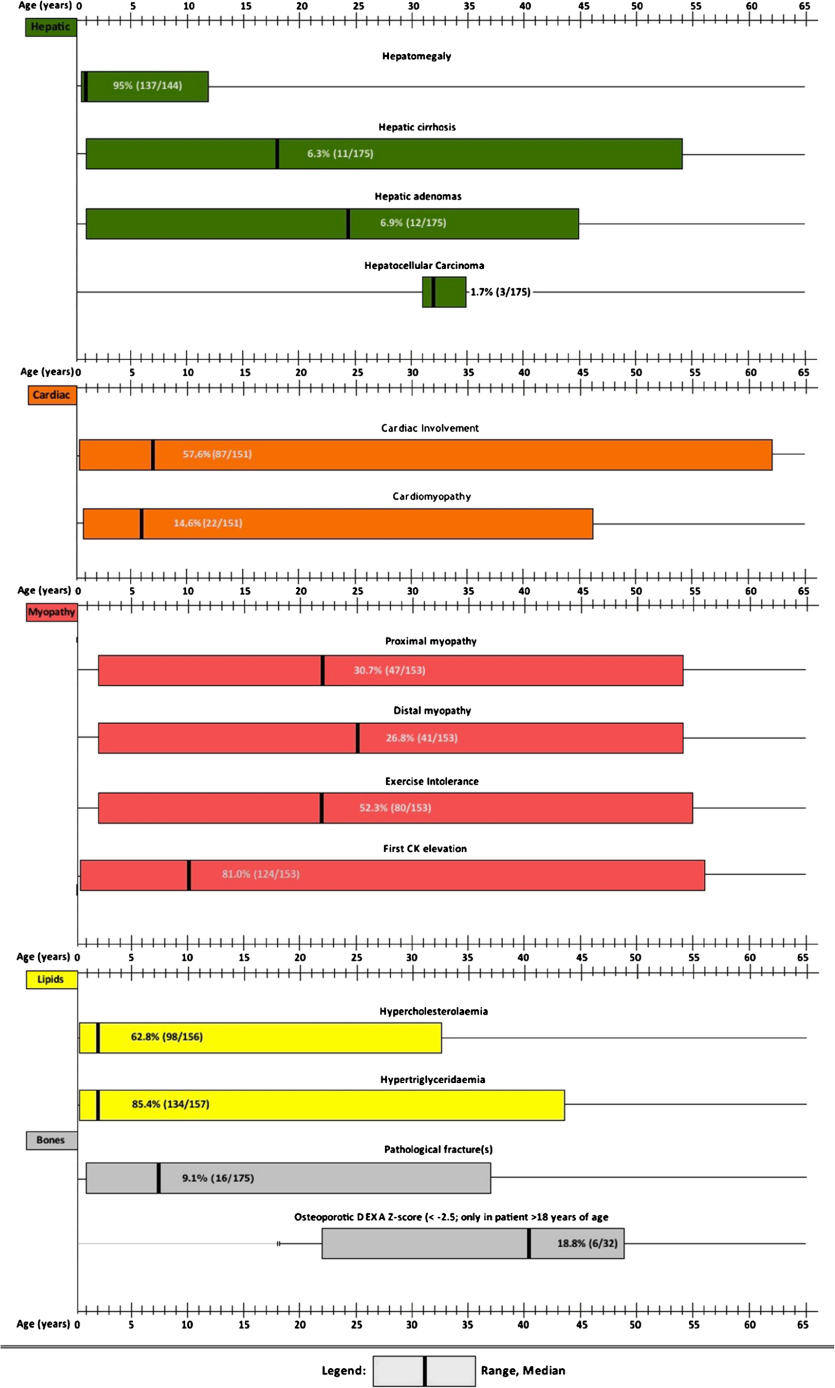
Age range of onset of disease features of GSDIII patients

Figure 2 presents the age (median and range) of onset of clinical features and complications.

#### Hepatic complications

The overall prevalence of severe hepatic complications (hepatic cirrhosis, adenomas and/or HCC) was 11 % (19 out of 175 patients; one GSDIIIb; 12 females). In 11 patients hepatic adenomas had been diagnosed (seven females), two of these patients were overweight at the time of diagnosis, and none of these patients used oral contraceptives. Alpha-fetoprotein levels were measured for eight patients, in none of these patients levels were over 40 ng/ml. In four patients with hepatic cirrhosis, orthotopic liver transplantation was performed at a median age of 32 years (15–35; three females; one GSDIIIb). In two of these patients (one GSDIIIb) hepatocellular carcinoma (HCC) was diagnosed postoperatively upon pathological examination. Three patients in total had been diagnosed with HCC, all of whom had progressed from hepatic fibrosis, to cirrhosis, and then HCC.

#### Cardiac complications

Cardiac involvement was reported in 58 % (87/151) of the GSDIIIa patients. Electrocardiographic and/or echocardiographic signs of left ventricular hypertrophy were found in 61 GSDIIIa patients. The remaining 26 patients had other forms of cardiac hypertrophy (isolated septal, right ventricular or biventricular hypertrophy). Cardiomyopathy was reported in 15 % (22/151) of all GSDIIIa patients. There were no laboratory parameters (cholesterol, triglycerides, ASAT, ALAT, CK) significantly increased in GSDIIIa patients with or without cardiomyopathy. Heart transplantations had not been performed in this cohort.

#### Neuromuscular complications

Muscular pain after minimal exercise was reported by 59 patients (34 %). Three patients were wheelchair dependent due to skeletal muscle involvement (signs of permanent muscle weakness with atrophy). Electromyography (EMG) data were available from 33 patients and mainly showed myopathic changes, such as fibrillations and positive sharp waves. In 124 GSDIIIa patients (81 %) CK concentrations were elevated at some point at a median age of 10 years (0.3 – 56.1). Cardiomyopathy was associated with a significantly higher prevalence of distal myopathy (Fisher’s exact p = 0.034) and muscular pain after minimal exercise (Fisher’s exact p = 0.030).

#### Growth and development

Adult height was reached at a median age of 19 years (15–23) (Table 2). There was no significant difference between GSDIIIa and GSDIIIb patients (p = 0.18). Of the patients who had reached adult height 24 % (21/86) had a BMI over 25 kg/m^2^ at the latest follow-up, i.e. overweight according to WHO criteria. Pubertal development was delayed in 36 patients, and there was no significant difference between the subtypes (p = 0.72). Furthermore, no significant difference was found in adult height in SDS between the patients who had delayed compared to normal pubertal development (p = 0.15).

**Table 2:** Height in SDS (compared to age-, gender- and ethnically corrected reference values) at latest follow-up in all patients

Age group (yrs)	N	Median height SDS (range)	N (%) < -2.0 SDS
0.0–2.0	10	−0.16 (-2.45–0.60)	1 (10)
2.0–5.0	20	−1.19 (-2.96–1.56)	2 (10)
5.0–10.0	27	−0.71 (-2.86–1.65)	5 (19)
10.0–15.0	24	−0.56 (-1.99–1.06)	–
15.0–20.0	26	0.26 (-1.81–2.50)	–
>20.0	61	0.13 (-2.86–2.46)	2 (3)
Total	168	−0.31 (-2.96–2.50)	10 (6)

#### Bones

Bone fractures were reported in 16 patients with median age 7.5 years (range 1-18). According to WHO criteria (T-scores), 14 GSDIIIa patients had normal bone mineral density (BMD), 12 patients had osteopenia and six patients had osteoporosis (all GSDIIIa patients). Significantly increased CK (Kruskal-Wallis p = 0.004) and ALAT (Kruskal-Wallis p = 0.027) values were observed in the osteoporosis group when compared with the normal BMD-group.

#### Hyperlipidaemia

Serum cholesterol and triglyceride concentrations were measured at the latest follow–up for 154 and 156 GSDIII patients, respectively. Cholesterol concentrations were consistently elevated (>5.17 mmol/L) in 34 % of the evaluated patients across different age groups. Triglyceride concentrations were mainly elevated (>1.69 mmol/L) in early childhood (in 23/29 79 %) patients up to 5 years. Between the age of 5 and 15 years this incidence decreased to 72 % (31/43), and after the age of 15 years stabilized to 40 % (34/84). Complications due to hyperlipidaemia, such as atherosclerosis or pancreatitis, were not reported in this population.

#### Endocrinologic complications

Type 2 diabetes mellitus (DM2) was diagnosed in eight out of 91 adult patients (9 %; all GSDIIIa), at median age of 38 years (32–44). Three patients were treated with insulin, and five with dietary adjustments. Four of these patients were overweight (BMI >25 kg/m^2^). HCC was not reported in patients with type 2 diabetes mellitus. Hirsutism was described in one patient. Irregular menses or amenorrhea was described in 19 patients. Polycystic ovaries were diagnosed by ultrasound in five patients at a median age of 19 years (6–30).

#### Mental and social development

Mental development was reported as normal in the majority of the patients, six patients displayed low or borderline intelligence (3 %) and one patient displayed severe developmental delay. Of the patients over 15 years of age (n = 101), 50 patients (50 %) had normal employment and 23 patients (23 %) were following normal general education. Sixteen patients (16 %) were unemployed, with eight of these patients being unemployed due to mental and/or physical disability. Thirty patients (21 females) had 64 healthy children.

#### Mortality

Three patients with GSDIIIa had died because of cardiomyopathy: one patient because of severe congestive heart failure at the age of 1 year; and two patients because of sudden cardiac death due to progressive cardiac fibrosis at the age of 29 and 39 years, respectively (Ramachandran et al 2012). One patient with GSDIIIa died at the age of 36 years because of severe liver failure due to end-stage hepatic cirrhosis in combination with hepatocellular carcinoma. One patient died of causes unrelated to his GSD.

### Dietary treatment

#### Dietary treatment at latest follow-up

Information regarding dietary treatment was collected on 171 patients (98 %; 87 children). Dietary restrictions were imposed on 16 patients; lactose restriction in ten patients and fat intake restriction in ten patients; 96 patients were reported to have a protein-enriched diet. In children (n = 49) the median cumulative protein intake was 2.9 grams per kilogram bodyweight per day (gr/kg/d; range 0.5–4.4), and in adults (n = 47) the median cumulative protein intake was 1.7 gr/kg/d (range 0.9–3.0). Impaired dietary compliance was reported by the treating physician and/or dietician in at least 29 patients (17 patients aged < 25 years).

#### History of dietary treatment

One hundred forty-six patients (83 %) were treated immediately after diagnosis. One hundred twenty-two patients were treated with uncooked cornstarch (UCCS) at some point; treatment with UCCS was stopped in 27 patients. Ninety-five patients were treated with UCCS at the latest dietary adjustment (64 patients age <18 years). Fifty-seven patients were treated with continuous gastric drip feeding (CGDF) at some point; CGDF was stopped in 18 patients. Thirty-nine patients were treated with CGDF at the latest dietary adjustment (33 patients age <18 years). In total 17 patients had received no dietary treatment at all (nine patients age <25 years).

## Discussion

ISGSDIII is a descriptive, retrospective, international, multi-centre cohort study of 175 patients. This study addresses important issues regarding clinical presentation and follow-up into adulthood.

Before discussing the results, some methodological issues need to be addressed. First, as ISGSDIII is a retrospective study, we have predominantly collected cross-sectional rather than longitudinal patient data, and there has been missing data. Unfortunately, IGSDIII did not collect data on fasting tolerance. Second, the ISGSDIII cohort is still relatively young, and follow-up has not extended into adulthood for all patients. This may have caused an underrepresentation of long-term complications. Third, as most participating centres and colleagues are centre of expertise, selection bias towards relatively severely affected patients may have affected the results. Fourth, patients with the extremely rare subtypes GSDIIIc (presumably the result of glucosidase debranching deficiency) and GSDIIId (presumably the result of transferase debranching deficiency) have not been included. Last, despite the use of a CRF, in different centres clinical and laboratory data are not yet recorded in a standardized and quantitative manner (for instance dietary parameters, echocardiographic parameters, quantification of skeletal muscle strength and exercise tolerance). Particularly the availability of dietary management data has been very limited. It additionally needs to be recognized that there may be a difference between prescribed diets and daily practice.

In contrast to the previous reports on GSDIII patients (Dagli et al 2010; Kishnani et al 2010; Laforêt et al 2012), ISGSDIII demonstrates that hypoglycaemia is a presenting symptom in just half of the patients. Therefore, in patients with a traditional clinical (hepatomegaly) and biochemical (elevated transaminase values, hyperlipidaemia) presentation, the diagnosis of GSDIII should not be rejected in the absence of (severe) hypoglycaemia. In addition, the finding of ketotic hypoglycemia after short fasting test is a major argument for GSDIII, as demonstrated recently (Hoogeveen et al 2015). Severe mental retardation and mortality due to metabolic derangement are uncommon in GSDIII patients. ESGSDI has reported high morbidity and mortality because of metabolic derangements with hypoglycaemia (Rake et al 2002). The difference between these studies may be partially explained by the relatively younger cohort of ISGSDIII. More importantly, there is a fundamental difference in metabolic compensation between GSDI patients (alternative lactate accumulates quickly, in the absence of ketones) and GSDIII patients (gluconeogenesis is intact and ketones can gradually be formed) during fasting.

Clear genotype-phenotype correlations are rare in GSDIII. The association between exon 3 mutations and GSDIIIb has been reported previously (Shen et al 1996; Elpeleg 1999; Shen and Chen 2002; Goldstein et al 2010). Interestingly, non-missense *AGL* mutations are overrepresented in the ISGSDIII cohort, whereas in most metabolic diseases missense mutations predominate. It can be hypothesized that missense mutations in the large *AGL* gene cause only minor reduction of GDE enzyme activity. Moreover, ISGSDIII demonstrates that GSDIIIa patients display a more severe clinical course than GSDIIIb patients. The latter group clinically presents at a later stage and has fewer complications, such as hepatic cirrhosis and HCC. The large ISGSDIII cohort did not identify additional correlations between *AGL* genotype and severe complications.

In accordance with previous reports (Vertilus et al 2010) ISGSDIII demonstrates that cardiac hypertrophy is common in GSDIIIa patients, mostly starting in the first decade of life (Fig. 2). Cardiac involvement remains stable over time in the majority of the affected patients, with even a portion of the patients regressing to normal values (data not presented). Hence, functional and clinically relevant hypertrophic cardiomyopathy is rare in GSDIIIa patients. Observations from GSDIIIa patients with severe hypertrophic cardiomyopathy suggest an important role of macronutrient intake (Derks and Smit 2015). To date it is speculative which macronutrient intervention is dominant, because each of the following has been described, i.e. decreased total caloric intake (Sentner et al 2012), increased protein intake (Dagli et al 2009; Sentner et al 2012), increased fat intake (Brambilla et al 2014), ketone bodies (Valayannopoulos et al 2011) and Atkins diet (Mayorandan et al 2014). It is not possible to draw causative conclusions from these single case observations, because increasing one macronutrient (either protein or fat) without affecting the other, inevitably affects the remaining macronutrient (carbohydrates). Based on at least two arguments it can be hypothesized that carbohydrate overtreatment may be an important risk factor for cardiac involvement and/or cardiomyopathy. First, decreased carbohydrate intake was the intervention shared by the above-mentioned reports in which cardiomyopathy resolved after dietary intervention. Secondly, decompensated structural cardiomyopathy is most frequently reported around the time of highest endogenous glucose requirements (i.e. childhood) and the prescription of relatively high amounts of dietary carbohydrate.

ISGSDIII demonstrates that GSDIII patients have (severe) growth retardation in (early) childhood, but eventually reach normal adult height. There is no significant difference in growth between GSDIIIa and GSDIIIb patients, suggesting that the metabolic demands on gluconeogenesis in GSDIII in general are more important than the presence of a muscular GDE deficiency.

ISGSDIII demonstrates a high incidence of bone fractures in paediatric GSDIII patients, suggesting the development of reduced BMD at an early age. The pathophysiology of reduced BMD is unclear, but an association with specific nutritional deficiencies in GSDIII (Folk and Greene 1984; Kishnani et al 1999), and reduced metabolic control in GSDI (Rake et al 2003) are mentioned. Recently, ALAT has been suggested to be a marker for metabolic control in GSDIII (Dagli et al 2010). ISGSDIII demonstrates a negative correlation between osteopenia/osteoporosis and ALAT, supporting the hypothesis that metabolic control affects BMD.

ISGSDIII reports a higher incidence of DM2 in ageing GSDIII patients than in the general population (i.e. 9 %: compared to 6 % in the general population in the western world according to the World Diabetes Foundation). Previous case reports and case studies have described the association between DM2 and GSDIII (Moe et al 1972; Oki et al 2000; Ismail 2009; Sharma 2009; Spengos et al 2009) but the aetiology is largely unknown. In the ISGSDIII cohort, half of the diabetic patients are obese, suggesting that decreased insulin sensitivity might play a role. Second, the constant intake of carbohydrate enriched nutrients to maintain euglycaemia may induce insulin resistance.

## Conclusions

ISGSDIII presents large heterogeneity between individual GSDIII patients. Most GSDIII patients present clinically in their first year of life with hepatomegaly as the major presenting symptom. From an acute disease in childhood, GSDIII develops into a chronic, progressive disease in adulthood, affecting liver, heart, skeletal muscle and bones. Chronic complications and the risk of developing DM2 emphasize the need of closely following the ageing GSDIII cohort. Standardized quantitative clinical data collection is warranted by an international longitudinal GSD patient registry and biobank.

## Electronic supplementary material

Below is the link to the electronic supplementary material.

ESM 1 (DOC 327 kb)
