# Supplementary material for: Glycogen storage disease type III: diagnosis, genotype, management, clinical course and outcome
Source: J Inherit Metab Dis. 2016 Apr 22;39:697–704. doi: 10.1007/s10545-016-9932-2 (PMC4987401; doi:10.1007/s10545-016-9932-2)
Supplement: Supplementary file 1 — (DOC 327 kb) [file 10545_2016_9932_MOESM1_ESM.doc]

*Questionnaire Nr.*

International Study

on

Glycogen Storage Disease

Type III

*___________________*

*ISGSDIII*

*By* : C.P. Sentner , MD/PhD Student

Department of Metabolic Diseases

Beatrix Children’s Clinic

University Medical Center Groningen

c.p.sentner@bkk.umcg.nl

*and*

G.P.A. Smit M.D., Ph.D., Professor in Paediatrics

Head of the Department of Metabolic Diseases

Beatrix Children’s Clinic

University Medical Center Groningen

g.p.a.smit@bkk.umcg.nl


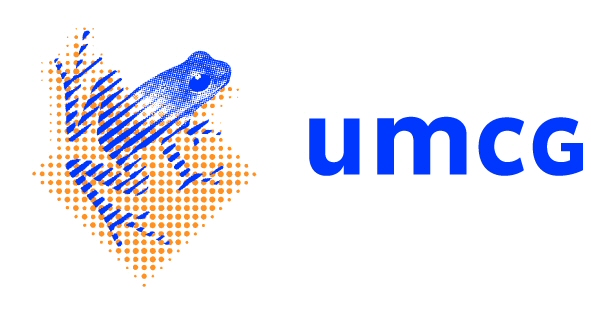


***International Study on Glycogen Storage Disease Type III Questionnaire***

__________________________________________________

| 0.1 | Investigator |  |
| --- | --- | --- |
| 0.2 | Date of completing this form | (ddmmyy) |

| 1.1 | Initials patient |  | | |
| --- | --- | --- | --- | --- |
| 1.2 | Date of birth | (ddmmyy) | | |
| 1.3 | Informed Consent | O no | O yes |  |

| 1.4 | Siblings with GSD III | O no | O yes |  |
| --- | --- | --- | --- | --- |
|  | If yes: initials and day of birth |  | | |

| 2.1 | Hospital |  |
| --- | --- | --- |
| 2.2 | Country |  |
| 2.3 | Patients doctor |  |

| 3.1 | Sex | O male | O female |  |
| --- | --- | --- | --- | --- |

| 4.1 | O alive | | | O deceased | |  | | |  | |
| --- | --- | --- | --- | --- | --- | --- | --- | --- | --- | --- |
|  | 4.2 | If alive | | | | | | | | |
|  |  | Present treatment in your hospital | | | | | | O yes | O no |  |
|  |  |  | If no: name hospital of present treatment : | | | | | | | |
|  |  |  | If yes : other hospital involved in treatment | | | | | | O no |  |
|  |  |  | | | | | O yes: name:  address: | | | |
|  | 4.3 | If deceased | | | Date of death (ddmmyy) | | | | | |
|  |  | Cause of death :  Based on clinical date :  Pathological findings: | | | | | | | | |

# 5 - Pregnancy and birth

| 5.1 | Complications in pregnancy | O no | O yes : |
| --- | --- | --- | --- |
| 5.2 | Complications at birth | O no | O yes : |
| 5.3 | Gestational age | Weeks | |
| 5.4 | Birth weight | Grams | |

# 6 - First symptoms

| 6.1 | First symptom | | | |
| --- | --- | --- | --- | --- |
|  | Hypoglycemia | | O no | O yes |
|  | Hepatomegaly | | O no | O yes |
|  | Developmental delay | | O no | O yes |
|  | Biochemical abnormalities | | O no | O yes, details: |
|  | Other | | O no | O yes, details: |
|  | 6.2 | At the age of | (ddmmyy ) | |
|  | 6.3 | At the age of | (ddmmyy) | |

# 7 - Diagnosis

| 7.1 | Clinical diagnosis | | | | **O** GSD III with myopathy | | | | | |
| --- | --- | --- | --- | --- | --- | --- | --- | --- | --- | --- |
|  | | | | | **O** GSD III without myopathy | | | | | |
| 7.2 | Based on Histology | | | | Liver biopsy | | | **O** yes | | **O** no |
|  |  | If yes | Date | | (mmyy) | | | | | |
|  |  |  | Name centre | |  | | | | | |
|  |  |  | | | Muscle biopsy | | | **O** yes | | **O** no |
|  |  | If yes | Date | | (mmyy) | | | | | |
|  |  |  | Name centre | |  | | | | | |
| 7.3 | Based on Enzymatic Diagnosis | | | | |  | | | | |
|  | Amilo-1.6 glucosidase deficiency in white blood cells | | | | | **O** yes | **O** no | | **O** not done | |
|  | Amilo-1.6 glucosidase deficiency in liver | | | | | **O** yes | **O** no | | **O** not done | |
|  | Amilo-1.6 glucosidase deficiency in muscle | | | | | **O** yes | **O** no | | **O** not done | |
|  | State subtype of GSD III (if tested) | | | | |  | | | | |
|  | Elevated RBC’s Glycogen | | | | | **O** yes | **O** no | | **O** not done | |
| 7.4 | Mutation analysis | | | State mutation if known : | | | | | **O** not done | |

# 8 - Dietary history & farmacological treatment

| 8.1 | Dietary history until the age of 25 years | Point out in **figure A** | | |
| --- | --- | --- | --- | --- |
| 8.2 | If age > 25 years: alterations in dietary treatment after the age of 25 | O no | | O yes |
|  | If yes : alteration : | | At the age of : | |
|  |  | | (years) | |
|  |  | | (years) | |
|  |  | | (years) | |
| 8.3 | Comments on dietary history : | | | |
| 8.4 | Farmacological treatment until the age of 25 years | Point out in  **figure A** | | |
| 8.5 | If age > 25 years: alterations in farmacological treatment after the age of 25 | O no | | O yes |
|  | If yes : alteration : | At the age of : | | |
|  |  | (years) | | |
|  |  | (years) | | |
|  |  | (years) | | |
| 8.6 | Comments on farmacological treatment: | | | |

**9 - Growth**

| 9.1 | Nationality | | **:** | | | | | | | | |
| --- | --- | --- | --- | --- | --- | --- | --- | --- | --- | --- | --- |
| 9.2 | Ethnic group | | O Asian | | O Caucasian | | | | | O Mediterrenean | |
|  |  | | O Negroid | | O other, nl : | | | | | | |
| 9.3 | Parental height | | Father: (cm) | | | | | Mother: (cm) | | | |
| 9.4 | Growth until the age of 23 years | 9.4.1 | Height (cm) | | | | | | Fill in **figure B** (physical – examination) | | |
|  | | 9.4.2 | Height (SD’s) ***** | | | | | |  |  |  |
|  |  | 9.4.3 | Weight (kg) | | | | | |  |  |  |
| 9.5 | Adult height | | (cm), reached at (age in years) | | | | | | | | |
| 9.6 | Pubertal development | | O not relevant, too young | | | | | | O unknown | | |
|  | | | O early ****** | | | O normal | | | | | O late ******* |
|  | If female, menarche at the age of | | (years) | | | | | O unknown | | | |
| 9.7 | Bone-age measurement | | O no | O yes | | |  | | | | |
|  | If yes, mention chronological and bone-ages: | | | | | | | | | | |

# * SD’s according to the standards of the country of origin.

# ** Before 8 years in girls or 9 years in boys.

*** After 14 years in girls or 15 years in boys.

# 10 - Hypoglycemia

| 10.1 | Admissions because of convulsions and/or coma due to documented hypoglycemia in each period | | | | | | | |
| --- | --- | --- | --- | --- | --- | --- | --- | --- |
|  | Age | Number of | age | Number of | age | Number of | age | Number of |
|  | 0-2 y |  | 2-5 y |  | 5-10 y |  | 10 + y |  |

#### 11 - Hyperlipidemia

| 11.1 | Blood cholesterol | Fill in **figure C** (laboratory investigations blood) | |
| --- | --- | --- | --- |
| 11.2 | Blood triglycerides |  |  |
| 11.3 |  | O no | O yes, at the age of (years) |
|  | If yes, course : | | |
| 11.4 | Atherosclerosis | O no | O yes, at the age of (years) |
|  |  | | O yes , at autopsy |
|  | If yes, course, symptoms and findings : | | |
|  |  |  |  |
|  |  |  |  |

#### 12 - Hepatic complications

| 12.1 | Cirrhosis | O no | | O yes, at the age of (years) | |
| --- | --- | --- | --- | --- | --- |
|  | Course : | | | | |
| 12.2 | Hepatic adenomas | O no | O yes, first detected at the age of  (years) | | |
|  |  | | O single one | | O multiple |
|  | Course : | | | | |

13 - Osteopenia

| 13.1 | Pathological fractures | O no | O yes, at the age of (years) |
| --- | --- | --- | --- |
|  | If yes, course: | | |

# 14 - Psychosocial

| 14.1 | Mental development | **O** low  {IQ < 65} | **O** borderline {IQ 65-85} | | | | **O** normal  {IQ 85-115} | | | | **O** high  {IQ>115} |
| --- | --- | --- | --- | --- | --- | --- | --- | --- | --- | --- | --- |
|  | Current school or most recent finished school : | | | | | | | | | | |
| 14.2 | If working – age | **O** unemployed | | |  | | | | | | |
|  | | **O** employed, profession : | | | | | | | | | |
| 14.3 | Married or long – standing relationship | **O** not relevant | | | | **O** no | | | **O** yes | |  |
| 14.4 | Children |  | | **O** not relevant | | | | **O** no | | **O** yes, number | |

##### 15 - Cardiomyopathy

| 15.1 | Abnormal Electrocardiogram | | O not assessed | O no | | O yes at the age of (years) | |
| --- | --- | --- | --- | --- | --- | --- | --- |
| 15.2 | Abnormal Echocardiogram | | O not assessed | O no | | O yes at the age of (years) | |
|  |  | Details: | | | | | |
|  |  | Progression | | | O no | | O yes |
| 15.3 | Clinical signs of heart failure | | O no | O yes at the age of (years) | | | |
| 15.4 | Requirement of medical therapy | | O no | O yes, started at the age of (years) | | | |
| 15.5 | Heart transplantation | | O no | O yes at the age of (years) | | | |
| 15.6 | Coronary heart disease | | O no | O yes at the age of (years) | | | |
| 15.7 | Angina pectoris | | O no | O yes at the age of (years) | | | |
| 15.8 | Myocardial infarction | | O no | O yes at the age of (years) | | | |

###### 16 - Neuromuscular

| 16.1 | Hypotonia in family | O no | | | O yes | | |
| --- | --- | --- | --- | --- | --- | --- | --- |
| 16.2 | Delayed walking * | O no | | | O yes | | |
| 16.3 | Proximal myopathy, muscular wasting | O no | | | O yes, at the age of (years) | | |
| 16.4 | Muscle pain | O no | | | O yes | | |
| 16.5 | Distal myopathy | O no | | | O yes, at the age of (years) | | |
| 16.6 | Exercise Intolerance | O no | | | O yes | | |
|  |  | Details: | | | | | |
| 16.7 | EMG (Electromyogram) | O not assessed | O normal | | | O abnormal at the age of | |
|  |  | Details: | | | | | |
| 16.8 | NCV (Nerve Conduction Velocity) | O not assessed | | O normal | | | O abnormal, details : |

* after 18 months of age.

17 - Endocrinology

| 17.1 | Diabetes mellitus / Insulin resistance | O no | O yes, if yes type : | |
| --- | --- | --- | --- | --- |
|  | | Age of onset (years) | | |
|  | | State initiated therapy: | | |
| 17.2 | Hirsutism | O no | | O yes |
| 17.3 | Irregular menses/Amenorrhea | O no | | O yes |
| 17.4 | Polycystic ovary by U.S. | O no | | O yes, at age of (years) |

18 - Renal Function

| 18.1 | Renal Function | O not assessed | O normal | O abnormal, details: | |
| --- | --- | --- | --- | --- | --- |
|  | | Age of onset (years) | | | |
| 18.2 | Tubular Dysfunction | O no | | | O yes, details: |
| 18.3 | Glomerular Dysfunction | O no | | | O yes, details: |
| 18.4 | Renal Tubular Acidosis | O no | | | O yes, state type: |
|  | | Age of onset ( years) | | | |
| 18.5 | Other Kidney abnormalities | O no | | | O yes, details: |

# Figure A Dietary history & farmacological treatment

Point out the dietary history (till the age of 25 years) in this figure.

Abbreviations used:

FM frequent meals

CS cornstarch added to the meals

GDF gastric drip feeding

TCS total cornstarch supplementation in grams per kilogram of bodyweight

TPS total protein supplementation in grams per kilogram of bodyweight

CS1 cornstarch once a night (f.e. before going to sleep)

CS2 cornstarch two times a night (f.e. before going to sleep and at 3.00 a.m.)

## H.P. high protein diet

F.T. farmacological treatment

| **Age (years)** | | **0.5** | **1** | **2** | **3** | **4** | **5** | **6** | **7** | **8** | **9** | **10** | **11** | **12** |
| --- | --- | --- | --- | --- | --- | --- | --- | --- | --- | --- | --- | --- | --- | --- |
| **Day** | FM |  |  |  |  |  |  |  |  |  |  |  |  |  |
|  | CS |  |  |  |  |  |  |  |  |  |  |  |  |  |
|  | GDF |  |  |  |  |  |  |  |  |  |  |  |  |  |
|  | TCS |  |  |  |  |  |  |  |  |  |  |  |  |  |
|  | TPS |  |  |  |  |  |  |  |  |  |  |  |  |  |
| **Nights** | FM |  |  |  |  |  |  |  |  |  |  |  |  |  |
|  | CS1 |  |  |  |  |  |  |  |  |  |  |  |  |  |
|  | CS2 |  |  |  |  |  |  |  |  |  |  |  |  |  |
|  | GDF |  |  |  |  |  |  |  |  |  |  |  |  |  |
|  | TCS |  |  |  |  |  |  |  |  |  |  |  |  |  |
|  | TPS |  |  |  |  |  |  |  |  |  |  |  |  |  |
| H.P. | |  |  |  |  |  |  |  |  |  |  |  |  |  |
| F.T. | |  |  |  |  |  |  |  |  |  |  |  |  |  |

| **Age (years)** | | **13** | **14** | **15** | **16** | **17** | **18** | **19** | **20** | **21** | **22** | **23** | **24** | **25** |
| --- | --- | --- | --- | --- | --- | --- | --- | --- | --- | --- | --- | --- | --- | --- |
| Day | FM |  |  |  |  |  |  |  |  |  |  |  |  |  |
|  | CS |  |  |  |  |  |  |  |  |  |  |  |  |  |
|  | GDF |  |  |  |  |  |  |  |  |  |  |  |  |  |
|  | TCS |  |  |  |  |  |  |  |  |  |  |  |  |  |
|  | TPS |  |  |  |  |  |  |  |  |  |  |  |  |  |
| **Nights** | FM |  |  |  |  |  |  |  |  |  |  |  |  |  |
|  | CS1 |  |  |  |  |  |  |  |  |  |  |  |  |  |
|  | CS2 |  |  |  |  |  |  |  |  |  |  |  |  |  |
|  | GDF |  |  |  |  |  |  |  |  |  |  |  |  |  |
|  | TCS |  |  |  |  |  |  |  |  |  |  |  |  |  |
|  | TPS |  |  |  |  |  |  |  |  |  |  |  |  |  |
| H.P. | |  |  |  |  |  |  |  |  |  |  |  |  |  |
| F.T. | |  |  |  |  |  |  |  |  |  |  |  |  |  |

# Figure B Physical Examination

Fill in date of examination, height in cm, height in standard deviation score (SDS, if known), weight in kg and liver in cm below the costal margin in the midclavicular line.

| Age (years) | ± 0.6 | ± 1.0 | ± 2.0 | ± 3.0 | ± 4.0 | ± 5.0 | ± 6.0 | ± 7 |
| --- | --- | --- | --- | --- | --- | --- | --- | --- |
| Date (ddmmyy) |  |  |  |  |  |  |  |  |
| Height (cm) |  |  |  |  |  |  |  |  |
| Height (SD’s)***** |  |  |  |  |  |  |  |  |
| Weight (kg) |  |  |  |  |  |  |  |  |
| Liver in m.c.l b.c.m |  |  |  |  |  |  |  |  |

| Age (years) | ± 8.0 | ± 9.0 | ± 10.0 | ± 11.0 | ± 12.0 | ± 13.0 | ± 14.0 | ± 15.0 |
| --- | --- | --- | --- | --- | --- | --- | --- | --- |
| Date (ddmmyy) |  |  |  |  |  |  |  |  |
| Height (cm) |  |  |  |  |  |  |  |  |
| Height (SD’s)***** |  |  |  |  |  |  |  |  |
| Weight (kg) |  |  |  |  |  |  |  |  |
| Liver in m.c.l b.c.m |  |  |  |  |  |  |  |  |

| Age (years) | ± 16.0 | ± 17.0 | ± 18.0 | ± 19.0 | ± 20.0 | ± 21.0 | ± 22.0 | ± 23.0 |
| --- | --- | --- | --- | --- | --- | --- | --- | --- |
| Date (ddmmyy) |  |  |  |  |  |  |  |  |
| Height (cm) |  |  |  |  |  |  |  |  |
| Height (SD’s)***** |  |  |  |  |  |  |  |  |
| Weight (kg) |  |  |  |  |  |  |  |  |
| Liver in m.c.l b.c.m |  |  |  |  |  |  |  |  |

# * SD’s according to the standards of the country of origin.

# Figure C Laboratory investigations blood

Fill in date of investigation, blood cholesterol, triglycerides, uric acid, haemoglobin, and hematocrit, mean corpuscular volume (MCV), calcium, creatinin, cholinesterase, alpha-fetoprotein, troponin, AST, ALT, creatine kinase, CK-MB, CK-MM and state which units are used in each investigation (e.g. mmo1/1).

| Age (years) | *units* | *± 0.6* | *± 1.0* | *± 2.0* | *± 4.0* | *± 6.0* | *± 8.0* | *± 10.0* | *± 12.0* |
| --- | --- | --- | --- | --- | --- | --- | --- | --- | --- |
| Date (ddmmyy) |  |  |  |  |  |  |  |  |  |
| Cholesterol |  |  |  |  |  |  |  |  |  |
| Triglycerides |  |  |  |  |  |  |  |  |  |
| Uric acid |  |  |  |  |  |  |  |  |  |
| Haemoglobin |  |  |  |  |  |  |  |  |  |
| Hematocrit |  |  |  |  |  |  |  |  |  |
| MCV |  |  |  |  |  |  |  |  |  |
| Calcium |  |  |  |  |  |  |  |  |  |
| Creatinin |  |  |  |  |  |  |  |  |  |
| Cholinesterase |  |  |  |  |  |  |  |  |  |
| Alpha-fetoprotein |  |  |  |  |  |  |  |  |  |
| Troponin |  |  |  |  |  |  |  |  |  |
| AST |  |  |  |  |  |  |  |  |  |
| ALT |  |  |  |  |  |  |  |  |  |
| Creatine Kinase (CK) |  |  |  |  |  |  |  |  |  |
| CK-MB |  |  |  |  |  |  |  |  |  |
| CK-MM |  |  |  |  |  |  |  |  |  |

| Age (years) | *± 14.0* | *± 16.0* | *± 18.0* | *± 20.0* | *± 22.0* | *± 25.0* | *± …...* | *± ….* | *± ….* |
| --- | --- | --- | --- | --- | --- | --- | --- | --- | --- |
| Date (ddmmyy) |  |  |  |  |  |  |  |  |  |
| Cholesterol |  |  |  |  |  |  |  |  |  |
| Triglycerides |  |  |  |  |  |  |  |  |  |
| Uric acid |  |  |  |  |  |  |  |  |  |
| Haemoglobin |  |  |  |  |  |  |  |  |  |
| Hematocrit |  |  |  |  |  |  |  |  |  |
| MCV |  |  |  |  |  |  |  |  |  |
| Calcium |  |  |  |  |  |  |  |  |  |
| Creatinin |  |  |  |  |  |  |  |  |  |
| Cholinesterase |  |  |  |  |  |  |  |  |  |
| Alpha-fetoprotein |  |  |  |  |  |  |  |  |  |
| Troponin |  |  |  |  |  |  |  |  |  |
| AST |  |  |  |  |  |  |  |  |  |
| ALT |  |  |  |  |  |  |  |  |  |
| Creatine Kinase (CK) |  |  |  |  |  |  |  |  |  |
| CK-MB |  |  |  |  |  |  |  |  |  |
| CK-MM |  |  |  |  |  |  |  |  |  |

Please return to:

Universitair Medisch Centrum Groningen

Beatrix Kinderziekenhuis

Secretariaat Metabole Ziekten

T.a.v. Chris Peter Sentner

Hanzeplein 1

9713 GZ Groningen

The Netherlands
